# Functionally interchangeable *cis*-acting RNA elements in both genome segments of a picorna-like plant virus

**DOI:** 10.1038/s41598-017-01243-z

**Published:** 2017-04-21

**Authors:** Jiangbo Guo, Junping Han, Junyan Lin, John Finer, Anne Dorrance, Feng Qu

**Affiliations:** 1 Department of Plant Pathology, Ohio Agricultural Research and Development Center, The Ohio State University, Wooster, OH 44691 USA; 2 Department of Horticulture and Crop Science, Ohio Agricultural Research and Development Center, The Ohio State University, Wooster, OH 44691 USA; 3 School of Mathematics, Physics and Biological Engineering, Inner Mongolia University of Science and Technology, Baotou, China; 4 Joint Genome Institute, Department of Energy, 2800 Mitchell Drive, Walnut Creek, CA 94598 USA

## Abstract

*Cis*-acting RNA structures in the genomes of RNA viruses play critical roles in viral infection, yet their importance in the bipartite genomes of the picorna-like, plant-infecting comoviruses has not been carefully investigated. We previously characterized SLC, a stem-loop structure in the 5′ untranslated region (UTR) of the bean pod mottle comovirus (BPMV) RNA2, and found it to be essential for RNA2 accumulation in infected cells. Here we report the identification of SL1, a similar *cis*-acting element in the other BPMV genome segment - RNA1. SL1 encompasses a portion of RNA1 5′ UTR but extends into the coding sequence for nine nucleotides, thus was missed in the previous study. While the stems of SL1 and SLC share little sequence similarity, their end loops are of the same size and identical for 11 of 15 nucleotides. Importantly, SL1 and SLC are functionally interchangeable, and separate exchanges of the stem and loop portions were likewise well tolerated. By contrast, the conserved loop sequence tolerated minimal perturbations. Finally, stem-loop structures with similar configurations were identified in two other comoviruses. Therefore, SL1 and SLC are likely essential comoviral RNA structures that play a conserved function in viral infection cycles.

## Introduction

The genomes of many single-stranded (ss), positive sense (+) RNA viruses fold into numerous secondary structures that play critical *cis*-acting roles in the infection cycles of these viruses^1^. The best known example of these RNA structures is probably the internal ribosomal entry site (IRES) element encoded within the 5′ termini of many picorna- and flavi-viruses, which enables efficient translation of virus-encoded polyproteins by guiding ribosomes directly to the start codon^2^. Long distance interactions between two or more secondary structure elements are also known to enhance cap-independent translation of viral genes, and to facilitate the synthesis of subgenomic RNAs of many viruses^3, 4^, whereas various tRNA-like elements at the 3′ termini of genomic RNAs function to prime the synthesis of (−) strand replicational intermediates^5^. Additionally, many internally encoded stem-loop structures were shown to exert diverse functions ranging from templating the synthesis of uridylylated VPg (viral protein genome-linked), anchoring viral RNA-dependent RNA polymerase (RdRP), and serving as the initiation site of genome encapsidation^6–8^.

Despite their recognized roles in viral multiplication cycles, RNA secondary structures have so far been studied in only a relatively small number of model viruses. Conversely, their broader prevalence remains largely unknown for the vast majority of (+) RNA viruses, including viruses of the plant-infecting *Comovirinae* subfamily (family *Secoviridae*, order *Picornavirales*). Comoviruses and animal viruses of the family *Picornaviridae* are considered to be evolutionarily related as the genomic RNAs of all these viruses encode large, undisrupted polyproteins that are proteolytically processed to release multiple mature viral proteins^9, 10^. Additional similarities between comoviruses and picornaviruses are: (i) they share similar, nonenveloped icosahedral particles; (ii) they harbor similar sized VPgs (between 22 and 28 amino acid [aa] long) that are much smaller than that of plant-infecting potyviruses (approximately 200 aa)^11^. However, while *cis*-acting RNA secondary structures have been systematically examined in poliovirus (PV) – a model picornavirus^12^, their involvement in comoviral infection cycles remain largely elusive.

**Figure 1:**
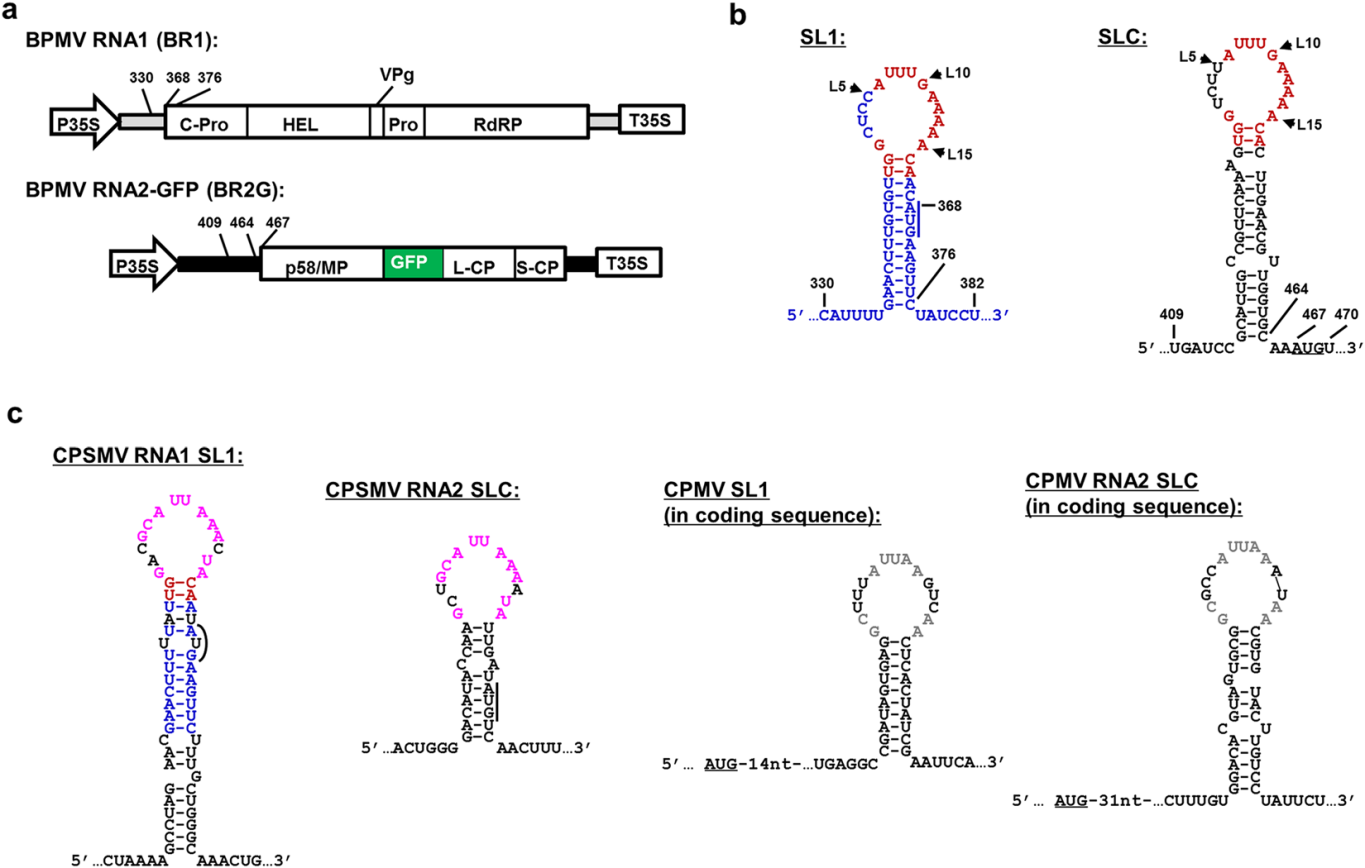
Genome organization of BPMV genomic RNA1 and 2, and the secondary structures of SL1 and SLC in three comoviruses. (**a**) Diagrams of constructs used to launch BPMV RNA1 (BR1) and RNA2 replication. The RNA2-GFP (BR2G) construct is a derivative of RNA2 cDNA with a GFP insert between MP and L-CP^15^. Both BR1 and BR2G cDNAs are flanked by the 35S promoter and terminator (P35S and T35S) of cauliflower mosaic virus to facilitate the transcription of viral RNAs by DNA-dependent RNA polymerase II of host cells. Mature viral proteins known to be processed from the polyprotein precursors are represented with varying sized boxes and the corresponding names. The terminal untranslated regions of BR1 and BR2G are depicted as thick gray and black lines, respectively. The numbers near 5′ UTR of BR1 and BR2G delimit the positions of SL1 and SLC, and their intimacy to the polyprotein start codons (also see B). (**b**) Secondary structures of SL1 and SLC. Nucleotides in blue and black are unique to SL1 and SLC, respectively. Those in red are conserved between SL1 and SLC, which are mostly located within the terminal loops. The AUG start codons of BR1 and BR2G polyproteins are underlined. Note that BR1 AUG is part of the stem of SL1. (**c**) Putative SL1 and SLC identified in RNA1 and RNA2 of CPSMV and CPMV. Loop nts shared by SL1 and SLC of CPSMV and CPMV are depicted in purple and gray letters, respectively. Note that CPSMV SL1 and BPMV SL1 share 11 base pairs within the stem (red and blue letters). Also note that both SL1 and SLC of CPSMV encompass the AUG start codons, whereas those of CPMV lie shortly downstream of the polyprotein start codons.

We have been interrogating the role of RNA secondary structures in comovirus infections using Bean pod mottle virus (BPMV) as a model^13^. BPMV is a bipartite comovirus with two single-stranded, (+) RNA segments, RNA1 and RNA2, that are both covalently linked to a VPg at the 5′ end, and polyadenylated at the 3′ end. Both RNA1 and RNA2, 6.0 and 3.6 kilobases (kb) in size, encode large polyproteins (Fig. 1a). All proteins required for viral RNA replication are processed from the RNA1-encoded polyprotein, including, from 5′ to 3′, a putative protease cofactor (C-Pro), an RNA helicase (Hel), VPg, a protease (Pro), and RdRP (Fig. 1a). RNA2 relies on RNA1 for its replication but encodes proteins required for the assembly of virus particles, viral cell-to-cell and systemic spread, as well as a protein (p58) required for RNA2 replication^14^ (Fig. 1a).

We previously reported the characterization of a stem-loop structure, designated SLC, within the 5′ untranslated region (UTR) of BPMV RNA2, and found it to be specifically required for the accumulation of BPMV RNA2 in infected cells^13^. SLC was initially presumed to be a structural element uniquely important for RNA2 replication, as its function was not restored by RNA1 5′ UTR replacement at the cognate location. Here we report the characterization of SL1, a novel hairpin structure within the 5′ terminus of BPMV RNA1 that is functionally exchangeable with SLC. We show that the stem portion of SL1 extends into the coding region of RNA1 polyprotein, contributing to the previous failure to complement SLC function with solely RNA1 5′ UTR. Importantly, both SL1 and SLC are required for the accumulation of their respective genome segments in infected cells, likely through their participation in the step of genome replication.

## Results

### An experimental system for interrogating SL1 and SLC

We previously reported the characterization of SLC, a stem-loop structure within 5′ UTR of BPMV RNA2 required for accumulation of RNA2 in infected cells^13^ (Fig. 1b). Based on the observation that the SLC-containing RNA2 5′ UTR could not be substituted by RNA1 5′ UTR, we initially speculated that SLC could be a *cis*-acting RNA element unique to RNA2 that facilitates RNA2 entry into virus replication complexes (VRCs) assembled by RNA1-encoded proteins^13^. However, upon closer inspection we were able to identify a novel stem-loop structure within RNA1 that spans from nucleotide number (no.) 336 to no. 376 (Fig. 1b, top left), with an undisrupted stem of 13 base pairs (bps), and a loop identical to SLC in size, sharing with SLC 11 of the 15 nucleotides (nts; Fig. 1b, red letters). Since the coding region of BPMV RNA1 begins at nt no. 368 (Fig. 1b, top left), this stem-loop structure, designated as SL1, extends from RNA1 5′ UTR into the first nine nts of the polyprotein-coding region. This led us to hypothesize that the previous failure to substitute RNA2 5′ UTR for that of RNA1 could be due to the incomplete replacement of SLC by SL1.

**Figure 2:**
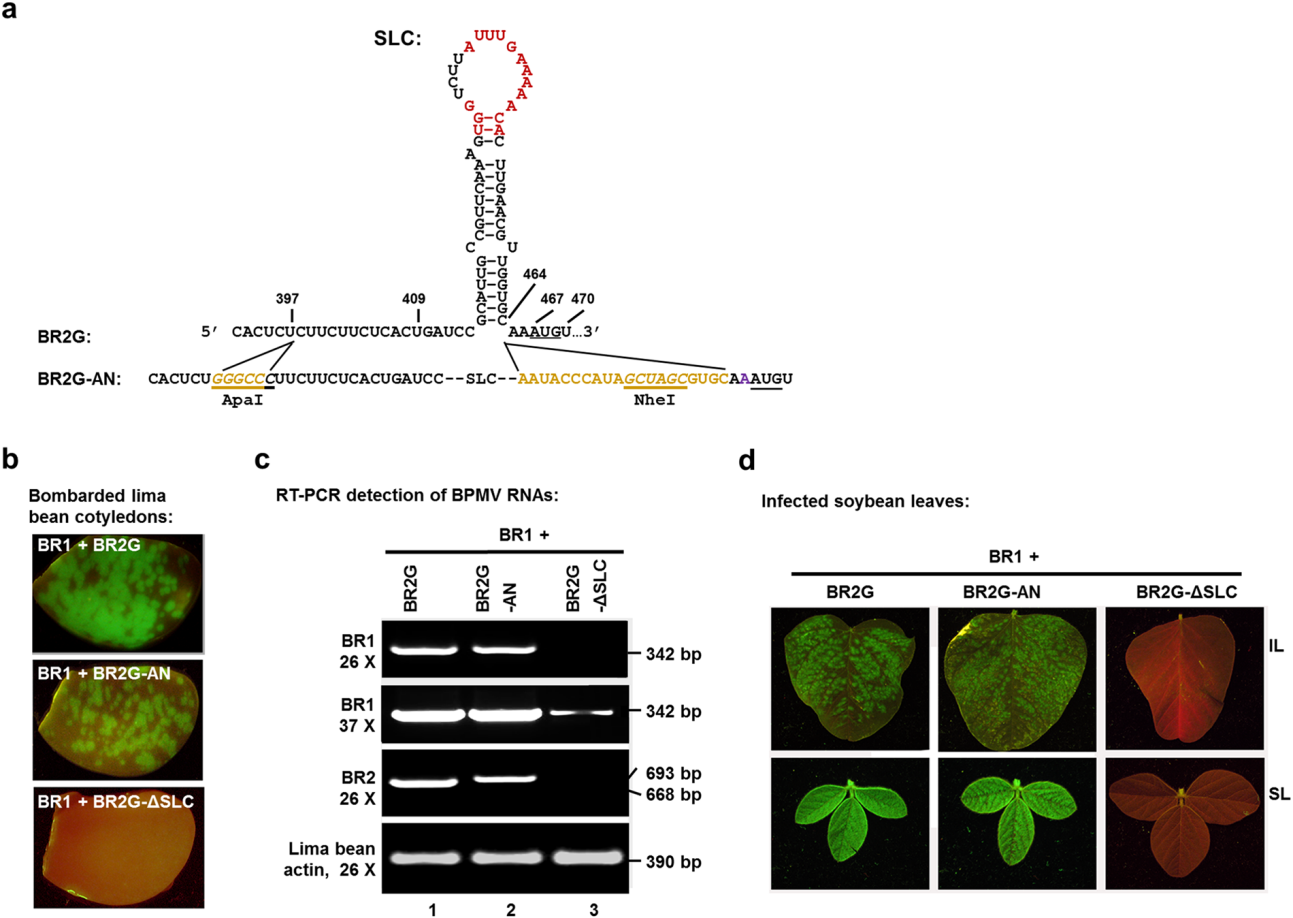
A BR2G derivative (BR2G-AN) that permits the manipulation of SLC sequence. (**a**) The sequence and structure of SLC and its vicinity, and modifications introduced to produce BR2G-AN. Specifically, five nts (GGGCC, in brown letters) were inserted between nt no. 397 and 398 to yield a new ApaI site (italic, underlined). Additionally, 20 extra nts (in brown letters) were inserted between nt no. 464 and 465 that also introduce a new NheI site (italic, underlined). Note that the first two of the 20 nts are identical to those immediately downstream of SLC in original RNA2 sequence, whereas the last four are duplicates of the last four stem nts. (**b**) Lima bean cotyledons bombarded with the construct mixes shown. (**c**) RT-PCR detection of both BR1 and BR2 RNAs in bombarded lima bean cotyledons. A 390-bp fragment of the lima bean actin mRNA was used as a control to ensure similar amounts of RNA were used in all reactions. (**d**) inoculated and systemically infected leaves of soybean plants (IL and SL) showing nearly identical infection efficiencies of BR2G and BR2G-AN.

To test this idea, we first engineered an RNA2 construct by flanking the SLC with two unique restriction enzyme sites – ApaI and NheI (Fig. 2a, underlined and italic letters. Brown letters represent newly inserted nts) to permit easy replacement of SLC. To ensure the folding of SLC and the translation of RNA2 polyproteins are minimally affected, we also separated SLC and the downstream NheI site with 10 extra nts (brown letters), and duplicated the last four nts (GUGC) of SLC downstream of the NheI site. These changes were made on BR2G, a GFP-expressing RNA2 derivative described in earlier studies^13–15^, resulting in BR2G-AN (Fig. 2a). Note that all constructs, including BPMV RNA1 (BR1), BR2G, BR2G-AN, as well as other constructs to be described later, are sandwiched by the 35S promoter and terminator (P35S and T35S) to permit DNA-launched initiation of BPMV replication (Fig. 1a).

We then compared BR2G-AN and BR2G side-by-side to ensure the infectivity of the former was not substantially compromised by the said manipulations. These constructs, together with BR2G-ΔSLC serving as a negative control, were mixed with BR1 and delivered into lima bean cotyledons through particle bombardment^13, 14, 16^. As shown in Fig. 1b, while the GFP foci on cotyledons bombarded with BR1 + BR2G-AN were slightly smaller than those on BR1 + BR2G cotyledons, the multiplication was sufficiently robust to permit subsequent analysis. This observation was also confirmed with strand-specific RT-PCR detecting (−) strand BPMV RNAs (Fig. 2c), as well as infections of soybean plants with extracts of lima bean cotyledons (Fig. 2d). Note that the level of BR1 was dramatically lower in the absence of a replicating RNA2 (Fig. 2c, top two panels, lane 3). In conclusion, BR2G-AN was suitable for additional studies aimed at replacing SLC with various mutated forms of SLC and SL1.

### SLC and SL1 are functionally interchangeable

**Figure 3:**
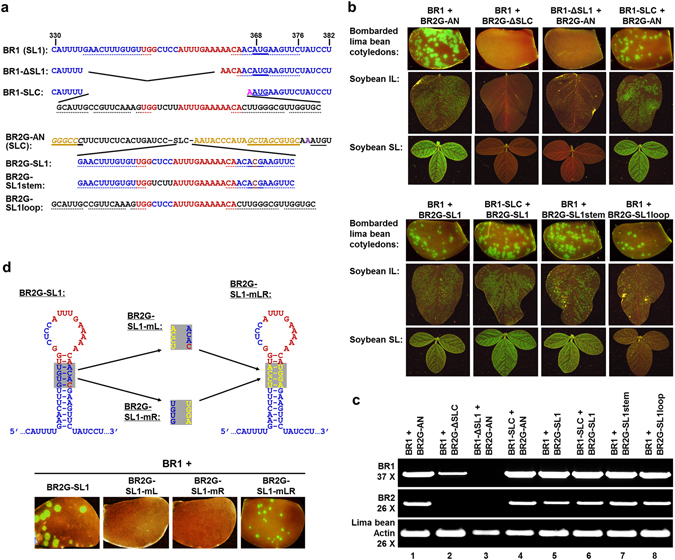
SL1 and SLC of BPMV are interchangeable. (**a**) Sequences of the SL1 and SLC portions of BR1 and BR2G-AN, respectively, and their deletion/replacement mutants. SL1 and SLC sequences are in blue and black letters, respectively; with the conserved loop nts in red. The nts involved in stems are underlined with dots. The AUG start codon in SL1, and its mutated form (ACG), are underlined with solid lines. Note that the last fifteen and nine nts of SL1 were retained in BR1-ΔSL1 and BR1-SLC, respectively, to preserve the AUG translational start codon. In BR2G derivatives containing SL1 segments, the AUG within the SL1 stem was changed to ACG to avoid its potential translational conflict with the RNA2 start codon downstream. (**b**) Lima bean cotyledons bombarded with various mutants, along with IL and SL images of soybean infected with the corresponding viruses. (**c**) RT-PCR detection of BR1 and BR2G RNAs in lima bean cotyledons bombarded with the mutants. (**d**) The stem of SL1 needs to remain base-paired in order for SL1 to be functional. Top: the predicted structure of SL1 in BR2G backbone, along with the three mutations (mL, MR, and mLR) introduced into the shaded area of the stem. Both mL and mR were predicted to disrupt the stem, whereas mLR should restore the base pairs with different sequences. Bottom: lima bean cotyledons bombarded with the constructs indicated above showing the restoration of replicability by the mLR mutant.

We next sought to determine whether SL1 was essential for RNA1 replication using the system described above. While deleting all 41 nts of SL1 was predicted to abolish the translation of RNA1 polyprotein, removing its first 26 nts (up to the last three nts of the loop) should not affect the coding capacity of RNA1 (BR1-ΔSL1 in Fig. 3a). Nevertheless, the BR1-ΔSL1 mutant was unable to accumulate BR1 RNA to levels detectable with our procedure (Fig. 3b, top half, third column; and Fig. 3c, top row, lane 3). Strikingly, this loss in RNA1 accumulation was largely reversed when the deleted region was replaced with RNA2 SLC sequence (BR1-SLC in Fig. 3a), as evidenced by the bright green fluorescent spots on bombarded lima bean cotyledons and the inoculated soybean leaves (IL), as well as evenly distributed GFP fluorescence on systemic soybean leaves (SL; Fig. 3b, top half, right hand columns). These results were also corroborated with RT-PCR detection of the (−) strands of BR1 and BR2 (Fig. 3c, top and middle rows, lane 4). We conclude that SL1 is essential for BPMV RNA1 accumulation, and its function could be complemented by SLC of RNA2.

To test reciprocally whether SL1 could also substitute for SLC in RNA2, the SL1 sequence was used to replace that of SLC in BR2G-AN (Fig. 3a, BR2G-SL1). Strikingly, the BR2G-SL1 was able to multiply in both lima bean cotyledons and inoculated as well as systemic leaves (IL and SL) of soybean plants, albeit at slightly lower levels than the BR2G-AN control (Fig. 3b, lower half, left column; Fig. 3c, lane 5). Furthermore, pairing BR1-SLC with BR2G-SL1 likewise resulted in robust BPMV infection (Fig. 3b, lower half, second column; Fig. 3c, lane 6). Therefore, SL1 and SLC are interchangeable stem-loop structures in RNA1 and RNA2, respectively, that function to promote the multiplication of respective genome segments. These results discount our previous model hypothesizing SLC as an RNA2-specific *cis*-element^13^.

SL1 and SLC share a highly conserved 15-nt loop with only the second to fifth nts (L1 to L5) differing from each other (Fig. 1b, compare the blue nts in SL1 with black nts in SLC). However, the stem portion of these two structures, though both predominantly double-stranded, share little sequence homology. To determine if stem and loop portions of each of the structure coordinate with each other, we then replaced the stem and loop portions of SLC separately with their counterparts in SL1 to create BR2G-SL1stem and BR2G-SL1loop (Fig. 3a). As shown in Fig. 3b (bottom half, right two columns) and Fig. 3c (lanes 7 and 8), while BR2G-SL1loop appears to be somewhat less efficient at facilitating BPMV replication and spread, both of these constructs nevertheless multiplied to readily detectable levels in both lima bean cotyledons and IL and SL of infected soybean plants. Therefore, the stem and loop portions of SL1 and SLC could be exchanged separately without abolishing their function in genome replication. These results are also consistent with our previous report showing that the stem of SLC tolerates considerable base changes as long as the base-paired nature is maintained^13^.

**Figure 4:**
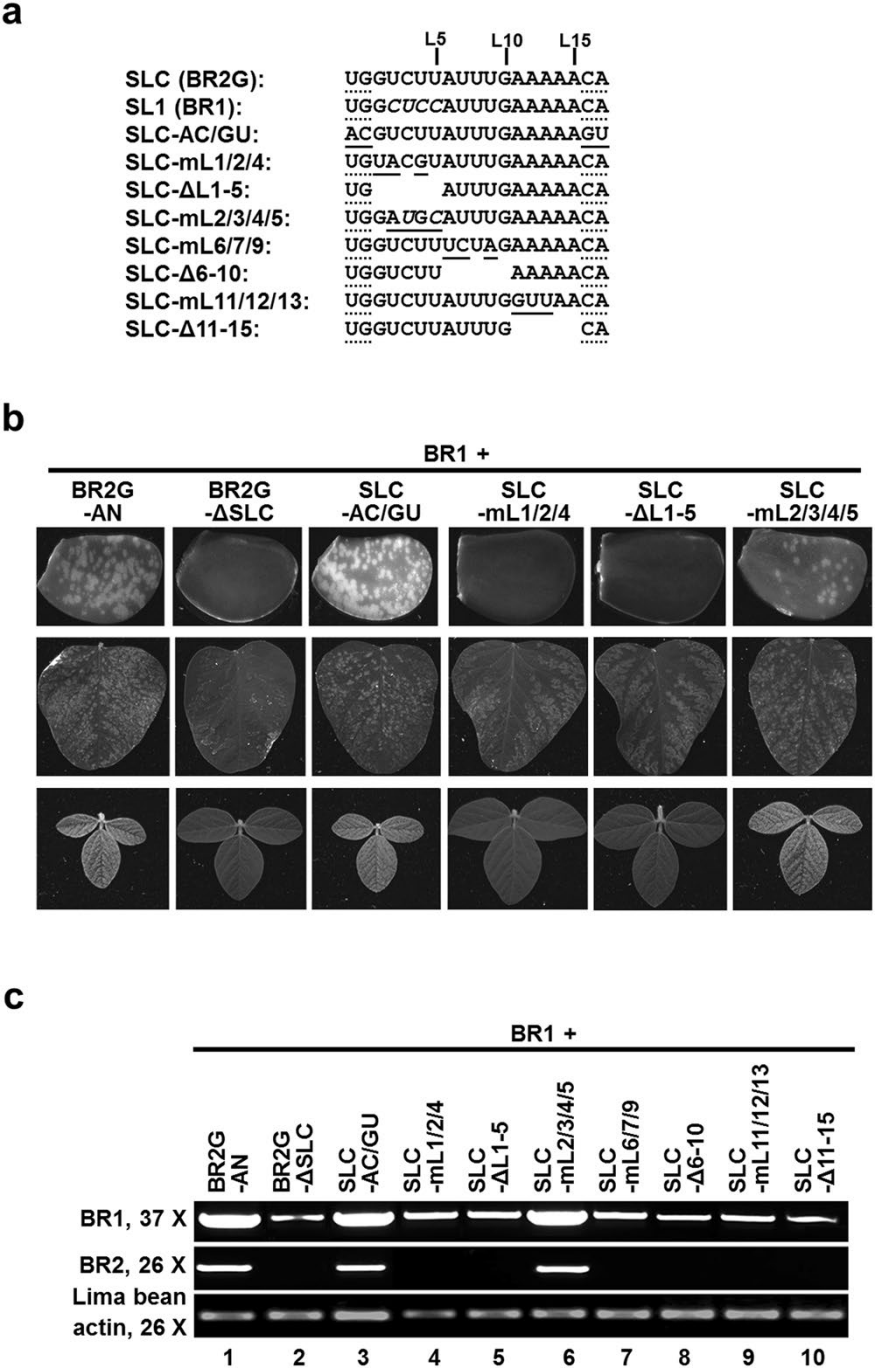
The loop portion of SL1 and SLC is highly conserved. (**a**) The nts within the loop of SLC and SL1 are shown along with the changes introduced in eight loop-specific mutants. The numbers above the nt sequences (L5, L10, L15) mark the positions of the corresponding nts in the loop. The nts at both ends, underlined with dots, make up the two neck base pairs adjacent to the loops. Mutated nts are highlighted with solid underlines, deletions are denoted by spaces. (**b**) The infectivity of a selected set of loop mutants in bombarded lima bean cotyledons (top row), inoculated (middle row) and systemic (bottom row) soybean leaves. (**c**) RT-PCR detection of the BR1 and BR2G viral RNAs in lima bean cotyledons bombarded with the loop mutants.

To further determine if the stem of SL1 needs to stay base-paired to maintain SL1 function, we generated three additional mutants, all of them in the BR2G-SL1 backbone (Fig. 3d). Specifically, the mL mutant replaced “GUGU”, four nts within the left side of the SL1 stem, with “UCCA”, so that the four shaded base pairs in Fig. 3d would be disrupted. Conversely, the mR mutant replaced “ACAC”, the four nts on the opposite side, with “UGGA”, disrupting the SL1 stem in a similar manner. Finally, the mLR mutant combined the changes in mL and mR so that the four SL1 base pairs would be restored with different primary nt sequence. As shown in the bottom half of Fig. 4d, both mL and mR mutants abolished RNA2 accumulation, as evidenced by a complete loss of green fluorescent spots on the bombarded lima bean cotyledons. Strikingly, the loss caused by mL and mR mutations were reversed by combining both mutations in the single mLR mutant, although the GFP spots were visibly smaller (Fig. 4d, bottom right). These results were further confirmed with RT-PCR (not shown), establishing that similar to the stem of SLC, that of SL1 also needed to remain base-paired in order to exert its function.

### The loop portion of SLC and SL1 is highly conserved

The 15-nt loops of SL1 and SLC are identical except for positions 2 to 5 (L2 to L5; Fig. 4a; also see Fig. 1b for conserved nts in red letters). In addition, the two stem base pairs immediately adjacent to the loops (G-C/U-A) are also identical in these two structures. To resolve the question of whether such sequence conservation is functionally important, we then carried out a systematic analysis of the loop sequence of SLC, in the BR2G-AN background. To first determine whether the identities of nts comprising the two neck base pairs are essential for SLC/SL1 function, we simultaneously changed the UG on the left side to AC, and the CA on the right side to GU to alter the nt identity while maintaining the base pairs. As shown in Fig. 4b (third row) and 4c (lane 3), the resulting SLC-AC/GU mutant replicated to levels similar to BR2G-AN. Therefore, the identities of nts at these positions could be altered if the base pairs are maintained. Although the need for these nts to remain double-stranded was not examined further, it was strongly inferred from these data, along with earlier results showing both SLC and SL1 required base-paired stems immediately below these nts (ref. 13 and previous section).

Three mutants were then created to assess the importance of the first five loop nts. The SLC-mL1/2/4 changed the L1, L2, and L4 nts (GUU in SLC, and GCC in SL1) to UAG (Fig. 4a). The SLC-ΔL1-5 mutant deleted the first five loop nts. Finally, the SLC-mL2/3/4/5 changed L2 to L5 (UCUU and CUCC in SLC and SL1, respectively) to AUGC. As shown in Fig. 4b (rows 4–6) and 4c (lanes 4–6), SLC-mL1/2/4 and SLC-ΔL1-5 completely abolished the infectivity of the mutant virus, whereas SLC-mL2/3/4/5 retained near wild-type level infectivity. These results revealed that (i) the nt identity of L1 (G) appears to be critical; (ii) the nt identities of L2-L5 tolerate a considerable level of variations; and (iii) the size of the loop appears to be invariable.

We further interrogated the role of L6-L15 by making both nt substitution mutations (SLC-mL6/7/9 and SLC-mL11/12/13) and deletion mutations (SLC-Δ6-10 and SLC-Δ11-15). As shown in Fig. 4c, lanes 7–10 (and data not shown), none of them was able to replicate to detectable levels. Accordingly, RNA1 replication was also lower as it was now restricted in single cells. These results suggest that the nt identity of L6-L15 tolerates little variations.

## Discussion

*Cis*-acting RNA structures play critical roles in the replication cycles of many (+) RNA viruses by regulating genome replication and transcription, viral protein translation, as well as the specificity of virion assembly^1–9^. However, the significance of such structures in the life cycle of bipartite comoviruses was not thoroughly investigated. Unlike other better studied (+) RNA plant viruses that rely on subgenomic RNAs for the translation of some more 3′ terminally located open reading frames, comoviruses adopt a gene expression strategy that involves the translation of long polyproteins from each of the two genome segments^17^. The polyproteins are then proteolytically processed to generate functional protein products. Another unique feature of comoviruses, but also of many animal viruses including polioviruses, is that the replication of their RNA genomes is primed by VPg, a small protein of fewer than 30 aas that is part of the RNA1-encoded polyprotein. VPg of animal-infecting picornaviruses is known to be uridylylated to become VPgpUpU. VPgpUpU then primes the synthesis of both (−) strand replicational intermediates from (+) strand genomes by pairing with the poly-A tail, and (+) strand progenies from (−) strand intermediates by pairing with two A residues at their 3′ ends^18^. It was further shown that the uridylylation of VPg depends on a stem-loop structure referred to as *cis*-acting element (CRE) that templates the addition of U residues to VPg by virus-encoded RdRPs^6, 18^.

It remains to be determined whether comoviral VPg is also uridylylated. Nevertheless, both SLC and SL1, of BPMV RNA2 and RNA1 respectively, bear striking resemblance to picornaviral CREs. SLC, an RNA stem-loop structure located immediately upstream of the coding sequence of BPMV RNA2, was identified by us in an earlier study^13^. Based on the fact that SLC lies within a region of RNA2 5′ UTR not exchangeable with RNA1 5′ UTR, we initially speculated that SLC might represent a structure unique to RNA2. However, this speculation has now been invalidated by the identification of SL1, a stem-loop in BPMV RNA1 that is structurally similar to SLC but requires a few nts at the beginning of RNA1 coding region for its integrity. Specifically, SLC and SL1 both have a 15-nt end loop, with 11 of the 15 nts being identical. Although the nt sequences of the SLC and SL1 stems are very different, they both form unbranched stems with few unpaired bases. The need for a base-paired stem in both SLC and SL1 was experimentally confirmed (ref. 13 and the current study).

Importantly, both SLC and SL1 are indispensible for productive multiplication of their respective genomic RNA segments, as their removal from the respective genome segments abolished accumulation of the corresponding genomic RNA. In light of their critical role, it is interesting to discover that these two stem-loop structures are mutually exchangeable – replacing SL1 with SLC, or vice versa, had minimal effect on viral RNA levels. We further demonstrated that the stem and the loop of these two structures could be separately exchanged without abolishing viral RNA accumulation. Together these results strongly suggest that SLC and SL1 play a critical, *cis*-acting role in the accumulation of the respective genomic RNA segments. Consistent with this assessment, the sequence of the 15-nt loop is highly conserved, with the nt identities of L1 and L6-15 being invariable.

What could be the exact function of SLC and SL1? One possibility is that they could help enhance the translation of the polyproteins encoded by BPMV RNAs. However, this seems unlikely as both wt and mL11/12/13 mutant forms of SLC were associated with similar levels of translation of a reporter gene (data not shown). Another possibility is that these stem-loop structures serve a role similar to that of CREs of animal-infecting picornaviruses. Several lines of evidence suggest that this is the case. First, the general structure of SLC and SL1 is very similar to that of CREs, in that they all have a long, unbranched stem and a large loop. In polioviruses, other enteroviruses, as well as rhinoviruses, the loops are mostly 14-nt in size, but can vary between 14 and 23 nts^18^. This suggests that the 15-nt loop size of SLC and SL1 is well within the range of variation. Another notable feature shared by the loops of SLC/SL1 and picornaviral CREs is that they all contain an invariable G at the first position, and an invariable A at the last position. Finally, all of the loops are enriched in A residues, presumably acting as templates for uridylylation.

Importantly, similar stem-loop structures can also be identified in both RNA segments of two other comoviruses – cowpea severe mosaic virus (CPSMV) and cowpea mosaic virus (CPMV). As shown in Fig. 1c, in CPSMV both SL1 and SLC partially overlap the polyprotein-coding sequences. Interestingly, SL1 of BPMV and CPSMV appear to have evolved from a common ancestor as their stems share a substantial level of sequence similarity, including ten identical base pairs (in blue and red letters), and a similar context of the AUG start codon. It is also interesting to note that while the loops of SLC and SL1 of the same virus share a high level of sequence conservation, those from different viruses are quite different, suggestive of convergent adaptation to a shared ligand (replication proteins?) in the same virus, but divergent evolution in different viruses.

Intriguingly, the CPMV SL1 and SLC appear to diverge further from BPMV and CPSMV. First, unlike SL1 and SLC of BPMV or CPSMV that encompass nts in the vicinity of translational start point, the CPMV SL1 and SLC are both located within the coding regions of RNA1 and RNA2 ORFs. It is also remarkable that the sizes of CPMV SL1 and SLC differ by one nt (15 nts for SL1 and 14 nt for SLC), which could be due to the fact that the same nts are involved in both RNA secondary structures and protein coding, thus are severely restrained in their evolution. Finally, compared with those of BPMV and CPSMV, the loops of CPMV SL1 and SLC also contained a greater degree of sequence variations.

To summarize, the evidence presented in this report suggests that SL1 and SLC of comoviruses are functionally analogous to CREs of picornaviruses. If this is true, the next question is why both genomic RNA segments of the bipartite comoviruses require a template-presenting structure for VPg uridylylation? It is possible that the VPgpUpU is generated only within the close vicinity of viral RNA, and immediately routed to the site of viral RNA replication, hence unavailable for use by a different genomic RNA segment. This would further suggest that the two genomic RNAs of comoviruses are replicated in separate replication complexes. In conclusion, our current study hints at the presence of CRE-like stem-loop structures in both genome segments of comoviruses. This work represents the first thorough examination of such structures in a comovirus. It advances our understanding of the mechanisms of comovirus replication, and should stimulate further investigations into these mechanisms.

## Methods

### Constructs

Constructs BR1, BG2G, and BR2G-ΔSLC have been described in previous studies^13, 14^. Constructs containing mutations within the SL1 and SLC regions were generated using PCR, with appropriate DNA oligonucleotides (sequences available upon request). The sequences of all mutants were confirmed with Sanger sequencing.

### Particle bombardment of lima bean cotyledons

Lima bean seeds (*Phaseolus lunatus* cv. Anderson Bush) were purchased from Earl May Seed and Nursery (Shenandoah, IA). Bombardment experiments were carried out following the procedure described by Hernandez-Garcia *et al*. (ref. 16).

### Strand-specific RT-PCR

Total RNAs were extracted from bombarded lima bean cotyledons at five days post bombardment, with a procedure described by Louine *et al*. (ref. 19), with minor modifications. After the quality of RNA was verified by UV spectrometry and agarose gel electrophoresis, approximately 5 µg RNA per sample was treated with TURBO DNA-free DNase according to the manufacturer’s instruction (Ambion, Austin, TX). Strand-specific reverse transcription was carried out with appropriate primers (available upon request), using the RevertAid reverse transcriptase (Thermo Bioscience). PCR was carried out using the EconoGreen PCR Master Mix (Lucigen, Middleton, WI).

### Fluorescence imaging and microscopy

The images of green fluorescent lima bean cotyledons and soybean leaves were captured using a Canon G6 camera equipped with a yellow filter, with the subjects placed under a long wave UV lamp. The bombarded cotyledons were also monitored on a daily basis with a fluorescence dissecting microscope (Model MZFLII, Leica, Heerbrugg, Switzerland).
